# Sex differences in cerebral blood flow and cardiac function in response to exercise in the heat

**DOI:** 10.1113/EP092955

**Published:** 2025-10-25

**Authors:** João Carlos Locatelli, Juliene G. Costa, Kristanti W. Wigati, Jesse L. Criddle, Julie J. Collis, Xingwei Xu, Louise H. Naylor, Howard H. Carter, Shane K. Maloney, Robert A. McLaughlin, Andrew Haynes, Helen Jones, Keith George, Daniel J. Green

**Affiliations:** ^1^ School of Human Sciences The University of Western Australia Crawley Western Australia Australia; ^2^ Medical Physiology and Biochemistry Department Faculty of Medicine, Universitas Airlangga Surabaya Indonesia; ^3^ Faculty of Health and Medical Sciences The University of Adelaide Adelaide South Australia Australia; ^4^ Research Institute for Sport and Exercise Sciences Liverpool John Moores University Liverpool UK

**Keywords:** females, global longitudinal strain, heat strain, males, middle cerebral artery velocity

## Abstract

We investigated the effect of exercising in hot conditions on cerebral blood flow and systolic left ventricular (LV) function in males and females, to explore sex differences. The experimental condition consisted of walking on a treadmill at 5 km/h and 2% incline, inside a heat chamber at 40°C (50% relative humidity), for 90 min. Middle cerebral artery velocity (MCAv) and LV global longitudinal strain (GLS) were assessed at baseline and every 30 min by means of transcranial Doppler and speckle‐tracking echocardiography, respectively. Thirty‐eight individuals (19♀, 19♂) were recruited. Both males and females exhibited non‐significant increases in MCAv from baseline at 30 min (♂ Δ = 2.55 ± 2.15, *P *> 0.05; ♀ Δ = 0.54 ± 2.53 cm s^−1^, *P *> 0.05; interaction *P *= 0.63). This was followed by a significant decrease at 60 (♂ Δ = −4.0 ± 1.23 *P *= 0.04; ♀ Δ = −5.41 ± 1.56 cm s^−1^, *P *= 0.03) and 90 min (♂ Δ = −6.08 ± 1.37 *P *< 0.01; ♀ Δ = −7.39 ± 1.40 cm s^−1^, *P *< 0.01). In males, there was a significant decrease in GLS from baseline at 60 (Δ = 2.17 ± 0.66%, *P *= 0.049) and 90 (Δ = 2.60 ± 0.77%, *P *= 0.036) min; no significant changes were observed in females. The correlation between changes in GLS and MCAv was higher for males (*r *= −0.631, *P *= 0.069) than for females (*r *= 0.252, *P *= 0.513). Males and females exhibited similar patterns of change in MCAv in response to a heat and exercise challenge. An exercise‐related reduction in GLS, and a higher correlation between changes in GLS and MCAv, were more apparent in males. These data suggest that sex differences may exist in the relationships between cerebrovascular and cardiac responses to exercise in the heat in humans.

## Abstract (highlights)

- **What is the central question of this study?**
  Do cerebrovascular and cardiac function responses differ between men and women who exercise in a hot environment?
- **What is the main finding and its importance?**
  While exercise in the heat had similar effects on cerebrovascular function in both males and females, males exhibited a reduction in global longitudinal myocardial strain, and a correlation between cerebrovascular and cardiac function. Under challenging physiological conditions, males and females differ in terms of their regulation of cerebrovascular function.

## 1 INTRODUCTION

The 2024 Lancet *Countdown on Health and Climate Change* report indicated that heat‐related mortality increased by 167% over the past two decades (Romanello et al., 2024), with ∼1.7 million deaths annually attributed to temperature variability between 2000 and 2019 (Wu et al., 2022). Thermoregulation in humans during exercise in hot environments presents a significant physiological challenge (Hunt et al., 2016), as blood flow must simultaneously support skeletal muscle metabolism, facilitate heat dissipation via the cutaneous circulation, and support motor cortex function (Rowell, 1974). These competing demands for blood flow pose a significant challenge to the cardiovascular system, as the combined vasodilator capacity of the skeletal muscle and skin vessel beds exceeds the maximal capacity for cardiac output (*Q̇*) (Rowell, 1974).

Although syncope during exercise in hot conditions is relatively uncommon, it has traditionally been assumed that, despite the challenges associated with cardiovascular regulation and blood flow distribution during exercise in the heat, cerebral blood flow (CBF) remains adequately regulated and maintained (Rasmussen et al., 2010). However, previous studies that have directly assessed changes in CBF during exercise in the heat have suggested that this may be an over‐simplification. For example, middle cerebral artery velocity (MCAv) was reduced (by 15%) during exercise in the heat, whereas no changes were apparent during normothermic exercise (Rasmussen et al., 2010). Gibbons et al. (2021) observed that CBF was reduced by up to 30% in response to exposure to a hot environment in healthy young adults. Other studies have suggested that redistribution of CBF occurs during exercise in the heat, with decreased blood flow through the internal carotid artery in the presence of increased extracranial skin blood flow (Sato et al., 2016). It has also been proposed that a reduction, or redistribution, of CBF during exercise in hyperthermic conditions may limit oxygen supply to the brain, challenging the homeostatic maintenance of cerebrovascular function (Secher et al., 2008). Indeed, lower CBF responses have been reported during heat and exercise exposure, compared to exercise in cool conditions at matched workloads (Périard & Racinais, 2015). These studies reinforce the fact that exercise in the heat induces changes in systemic physiological variables and cerebrovascular responses that are inter‐related.

Despite an evidence‐base that differences exist between males and females in terms of the physiological responses to exercise and thermoregulation (Gagnon & Kenny, 2012; Wickham et al., 2021), few studies have directly addressed sex differences in CBF responses when exercising in hot conditions. The majority of research has been conducted in males, with findings frequently extrapolated to females rather than examined explicitly (Fernández‐Peña et al., 2023). Of the few studies available, Rivas et al. (2020) reported that, while a reduction in MCAv occurs when exercising in the heat in both sexes, the effect was larger in females. The authors speculated that differences in cardiovascular function and control may be responsible for these apparent sex differences, although responses such as left ventricle (LV) global longitudinal strain (GLS) were not assessed in this study (Rivas et al., 2020). Other factors such as CO_2_ reactivity, cerebral autoregulation and vascular coupling also play an important role in the regulation of CBF (Ogoh, 2019), with apparent sex differences such as evidence that females have a lower ventilatory reactivity than males (Tallon et al., 2020). However, increases in *Q̇* during exercise also affect CBF and diminished capacity to increase *Q̇*, which occurs with ageing and in the presence of cardiovascular diseases, prevents adequate blood supply to the brain during exercise (Ogoh, 2025). Based on the study of Rivas *et al.* (2020), who speculated that females would undergo a greater cardiovascular strain to regulate body temperature and maintain adequate cerebral perfusion, we hypothesised that females would present a larger reduction in LV GLS. GLS is a novel and highly sensitive marker of systolic LV function which detects preclinical changes in systolic function, even in the presence of preserved ejection fraction (Karlsen et al., 2019). The assessment of GLS therefore provides novel information regarding changes in systolic function during exercise in the heat, as well as being a potential upstream mediator of cerebral perfusion.

Consequently, we investigated the acute effects of exercise in the heat on MCAv and LV GLS in healthy, adult males and females to explore possible sex differences, as well as the association between LV function and cerebrovascular function. Our hypotheses were that: (1) both males and females would exhibit reduced MCAv and LV GLS during exercise in the heat; (2) females would present a larger decrease in MCAv and GLS than males; and (3) the relationship between cardiac and cerebral responses would differ between males and females.

## 2 METHODS

### 2.1 Ethical approval

This study was conducted in accordance with the 1964 *Declaration of Helsinki* and its later amendments, except for registration in a database. The study was approved by the Human Research Ethics Committee of the University of Western Australia (Ref: RA/4/20/5716). The participants provided written informed consent prior to the commencement of the study.

### 2.2 Study design

In the present study, each participant visited the laboratory twice within 7 days. Inclusion criteria included both male and female individuals (18–35 years) who were capable of exercising on a treadmill, had no history of cardiovascular, cerebrovascular, metabolic, respiratory or musculoskeletal disorders, were non‐smokers, and had no use of cardiovascular medication. The participants were asked to relate their sex assigned at birth (e.g. male or female), and also to describe their gender (‘Man', ‘Woman’, ‘Non‐binary’, ‘[I/they] use a different term’, or ‘Prefer not to answer’). Every individual reported their gender as consistent with the sex that was recorded at birth.

### 2.3 Baseline assessments – Visit 1

Anthropometric, body composition and aerobic capacity (${\dot{V}}_{O_{2}\max}$) were assessed at an initial testing session, with the experimental condition undertaken within the subsequent 7 days. For both laboratory visits, participants were asked to fast overnight and to refrain from moderate‐to‐vigorous exercise, alcohol and caffeine consumption for 24 h. Both baseline and experimental condition visits were conducted at the same time of the day, between 07.00 and 10.00 h, for all participants.

Body mass was measured using an electronic scale (CPW plus – 300, ADAM Equipment, Milton Keynes, UK) with 0.1 kg precision. Participants were barefoot and wore light clothing. Height was measured using a wall‐coupled stadiometer (SECA, Hamburg, Germany) with 0.01 m precision. Body composition was assessed via dual‐energy X‐ray absorptiometry (Lunar iDXA, GE Healthcare, Chicago, USA). Total body and segmental indices of lean mass, fat mass and bone mineral density were calculated. A treadmill incremental exercise test was used to assess aerobic capacity (${\dot{V}}_{O_{2}\max}$) during which the speed and gradient increased every 3 min. Once the speed reached 8.0 km h^−1^ and the gradient 10%, the latter was fixed until the end of the test, while the speed increased 1.0 km h^−1^ every 3 min until volitional exhaustion. Verbal encouragement was given to stimulate maximal effort. Heart rate (HR; Polar H10 HR monitor, Polar Electro Oy, Kempele, Finland) and rate of perceived exertion (RPE) were recorded at the end of every stage (i.e. last 15 s of each stage). Respiratory measures such as expiratory flow and expired concentrations of O_2_ and CO_2_ were measured continuously using a calibrated metabolic measurement system (Parvomedics TrueOne 2400, Salt Lake City, UT, USA).

### 2.4 Experimental session – Visit 2

The experimental protocol consisted of walking for 90 min on a treadmill set at 5 km h^−1^ and 2% gradient inside a temperature‐controlled chamber set to 40°C and 50% relative humidity (RH). Water was available to the participants ad libitum during the experimental session (the mean water consumption was 0.84 ± 0.65 L). The participants were asked to ingest a radiotelemetry gastrointestinal pill (eCelsius Performance; BodyCap Medical, Hérouville‐Saint‐Clair, France) 6–7 h prior to the experimental session to assess core body temperature (*T*_c_) (Byrne & Lim, 2007). Pre‐experimental session assessments were performed outside the chamber, and participants started exercise immediately after entering the chamber.

Close attention was paid to participant well‐being throughout the exercise protocol. The exercise was immediately stopped under participants' request, and/or if they presented any heat‐related symptoms (e.g. dizziness, light‐headedness, weakness, confusion, headache, nausea), or if their *T*_c_ exceeded 39.0°C. Cerebral blood flow velocity, echocardiographic, and respiratory measurements were acquired before the commencement of the experimental protocol and then every 30 min (i.e. at baseline [*t*_0_], 30 [*t*_30_], 60 [*t*_60_], and 90 [*t*_90_] min after the commencement of exercise).

### 2.5 Cerebral blood flow velocity

Middle cerebral artery velocity (MCAv) was assessed using a 2‐MHz ST3 Transcranial Doppler (TCD) ultrasound system (Spencer Technologies, Seattle, WA, USA). With participants lying in the supine position, probes were positioned bilaterally on each transtemporal acoustic window and fixed using a head frame, which was kept on participants’ heads throughout the whole experimental protocol. The MCAv signal was identified using depth, velocity and waveform as references, as described elsewhere (Aaslid et al., 1982). Data on MCAv were acquired for 10 min before the commencement of the exercise session, with participants outside the chamber, and during the last 10 min of each 30‐min exercise period (i.e. *t*_30_, *t*_60_, and *t*_90_) with participants walking on the treadmill within the chamber. MCAv was collected using PowerLab and exported in raw format to LabChart (LabChart 8; ADInstruments, Bella Vista, NSW, Australia) for subsequent analysis. Mean values of the last 5 min of each 10‐min assessment period were calculated and used for analysis.

### 2.6 Echocardiographic assessments

Cardiac function was assessed via a transthoracic echocardiogram using a commercially available ultrasound system (EPIQ CVx; Philips Ultrasound, Andover, MA, USA) using a X5‐1 MHz transducer, performed by an experienced and accredited sonographer in accordance with comprehensive guidelines (Mitchell et al., 2019). A baseline transthoracic echocardiogram was performed prior to commencement of the exercise session (*t*_0_), with participants outside the chamber, and repeated at *t*_30_, *t*_60_ and *t*_90_ after exercise commencement within the chamber. Echocardiographic images were obtained with the participants in the semi‐recumbent left lateral position at end expiration, with the transducer angled to obtain orthogonal views of the left ventricle (LV). Images were optimised prior to acquisition utilising transmit frequency, greyscale maps, gain, and by minimising depth and sector width to optimise the frame rates. Three complete cardiac cycles were obtained for every image. Images were digitally stored in cine‐loop DICOM format and transferred to the Philips Ultrasound Workspace (TOMTEC Imaging Systems GmbH, Freisinger Strasse, Unterschleissheim, Germany) for offline *post hoc* analysis. Stroke volume (SV) was calculated as the difference between LV end‐diastolic volume and end‐systolic volume. *Q̇* was calculated by multiplying SV by HR. Global longitudinal strain (GLS) was assessed by 2D speckle tracking echocardiography (STE) (Negishi et al., 2015). Mean values from the 18 segments obtained from the apical four, three and two‐chamber views were used to calculate GLS. An echocardiographic examination bed was positioned inside the chamber and beside the treadmill, wherein, at the end of each 30‐min period, the participants were instructed to cease exercise and immediately transition to the bed and lie in the same semi‐recumbent position for rapid echocardiographic assessment, which took ∼2.5 min. Thereafter, exercise resumed.

### 2.7 Skin blood flow

Skin blood flow (SkBF) was assessed by means of laser Doppler flowmetry. Doppler array laser probes (model 413, Periflux 500 system; Perimed, Jarfalla, Sweden) were placed at the right proximal forearm distal to the decubital fossa and at the right side of the back above the scapula. The probes were fixed using double‐sized ring‐shaped adhesives and Fixomull tape to avoid displacement. SkBF was collected with participants walking on the treadmill for the last 10 min of every 30‐min exercise period, being calculated as the mean SkBF of forearm and back‐derived values and was expressed in perfusion units (PU).

### 2.8 Respiratory measurements

Resting expired respiratory concentrations of O_2_ and CO_2_ were measured for 10 min using a calibrated metabolic measurement system (Parvomedics TrueOne 2400, Salt Lake City, UT, USA) with participants lying in the supine position in a temperature‐controlled room set at 24°C. The participants were set up with a mouthpiece and instructed to breathe only through their mouth. A nose peg was placed on participants’ noses to ensure blockage of the nasal airway. The measurements were repeated during the last 10 min of every 30‐min exercise period with the reinsertion of the mouthpiece and nose peg. Mean values of end‐tidal CO_2_ ($P_{\mathrm{ETC}O_{2}}$), ventilation and respiratory rate were calculated. Despite familiarisation with the mouthpiece and prior completion of ${\dot{V}}_{O_{2}\max}$ tests utilising the same equipment, some participants nonetheless felt uncomfortable with the mouthpiece and nose peg during exercise in the heat, and asked to have them removed. We did not include respiratory data in these individuals, resulting in lower sample sizes for some variables.

### 2.9 Statistical analysis

Continuous variables are expressed as mean and standard deviation, unless otherwise stated. Data were analysed using the SPSS Statistics (Version 29) (IBM Corp., Armonk, NY, USA). Normal distribution of data was checked via the Shapiro–Wilk test. Sex differences at baseline were assessed by applying an unpaired Student's *t*‐test. For the variables that presented sex differences at baseline, we used ANCOVA for sex comparisons at *t*_30_, *t*_60_ and *t*_90_, using baseline values as covariates. Mixed models analysis of variance (ANOVA) was performed to identify the effect of time and sex, and their interaction, on each variable. Within‐group differences regarding time were analysed by applying the one‐way repeated measures ANOVA with Bonferroni correction. Pearson's correlation was performed to identify significant correlations between the changes in the respective variable from baseline to *t*_90_. The significance level was set at *P* ≤ 0.05.

## 3 RESULTS

### 3.1 Participants

Thirty‐eight individuals (19♂ 19♀) were initially recruited, and 19 males and 17 females completed 90 min of the experimental protocol (Table 1); two participants experienced heat‐related symptoms (i.e. light‐headedness and nausea) and discontinued exercise. Males and females were matched for body mass index, but males were younger, taller and heavier than females. The males also had significantly more lean mass and less fat mass (%), higher ${\dot{V}}_{O_{2}\max}$, SV, cardiac output (*Q̇*), $P_{\mathrm{ETC}O_{2}}$ and lower *T*_c_ than the females.

**TABLE 1:** Participants’ characteristics at baseline.

Characteristic	Males (*n* = 19)	Females (*n* = 19)	*P*
Age (years)	25.74 ± 3.62	30.00 ± 6.26	**0.016**
Height (cm)	181.3 ± 7.8	169.4 ± 7.1	**<0.001**
Body mass (kg)	74.57 ± 10.53	62.13 ± 5.11	**<0.001**
Body mass index (kg m^−2^)	22.84 ± 2.58	21.65 ± 1.77	0.112
BSA (m^2^)	1.93 ± 0.17	1.71 ± 0.09	**<0.001**
Body composition			
Fat mass (kg)	14.79 ± 5.98	17.36 ± 4.96	0.177
Fat mass (%)	19.49 ± 6.39	29.19 ± 7.03	**<0.001**
Lean mass (kg)	57.01 ± 7.73	39.91 ± 7.68	**<0.001**
Lean mass (%)	76.19 ± 6.15	64.80 ± 11.10	**<0.001**
Cardiorespiratory fitness			
${\dot{V}}_{O_{2}\max}$ (mL kg^−1^ min^−1^)	58.65 ± 11.87	41.40 ± 9.46	**<0.001**
Body temperature			
Body core temperature (°C)	36.94 ± 0.24	37.21 ± 0.32	**0.005**
Cerebral blood flow velocity			
MCAv (cm s^−1^)	59.64 ± 12.06	61.62 ± 8.92	0.638
Skin blood flow (PU)	23.59 ± 7.49	23.00 ± 10.62	0.849
Cardiac parameters			
Heart rate (bpm)	63 ± 12	66 ± 15	0.536
Global longitudinal strain (%)	−20.11 ± 2.45	−21.72 ± 2.74	0.081
Stroke volume (mL)	83 ± 20	68 ± 13	**0.011**
Cardiac output (L min^−1^)	5.2 ± 1.18	4.23 ± 0.76	**0.009**
Respiratory markers			
Ventilation (L min^−1^)	8.94 ± 2.02	8.59 ± 2.43	0.673
Respiratory rate (bpm)	11.29 ± 3.62	13.00 ± 3.40	0.199
$P_{\mathrm{ETC}O_{2}}$ (mmHg)	26.70 ± 3.77	23.66 ± 1.72	**0.014**

Data are presented as mean and standard deviation. BSA, body surface area; MCAv, Middle cerebral artery velocity; $P_{\mathrm{ETC}O_{2}}$_,_ end‐tidal CO_2_ partial pressure; ${\dot{V}}_{O_{2}\max}$ maximal oxygen uptake.

### 3.2 Thermoregulatory, haemodynamic and respiratory responses to exercise in the heat

**FIGURE 1:**
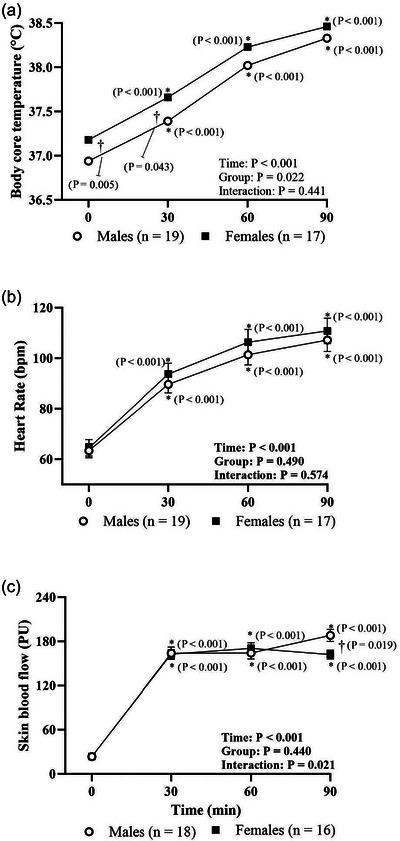
(a) Body core temperature; (b) heart rate and; (c) skin blood flow during the exercise protocol. ^*^Difference from baseline; ^†^difference between groups. Data are presented as means and standard error.

Throughout the exercise + heat protocol, the *T*_c_ increased significantly in both sexes (*P *< 0.001 for all time points and both sexes), with a significant difference between the groups (*P *= 0.022) wherein the females presented a higher body core temperature at *t*_0_ (*P* = 0.005) and *t*_30_ (*P* = 0.043) than males. However, when male and female core temperature responses were normalised as changes from baseline, there were no sex differences at *t*_30_ (*P* = 0.627), *t*_60_ (*P* = 0.853) or *t*_90_ (*P* = 0.447). No interaction between time and group (*P *= 0.441) was observed (Figure 1a). A significant and similar increase in HR was observed for males and females at all time points in relation to baseline values (*P *< 0.001 for all time points), but there was no difference between the groups at any time point (Figure 1b).

Complete data sets on SkBF were collected in 34 participants (18♂ 16♀). In two participants, data were lost due to Doppler probe issues due to excessive sweating. A significant time × group interaction was observed for SkBF (*P *< 0.021), due principally to a higher SkBF at *t*_90_ in males than females (*P* = 0.019). Males and females both had an increase in SkBF at *t*_30_, *t*_60_ and *t*_90_ in comparison with baseline values (*P *< 0.001 for all time points) (Figure 1c).

**FIGURE 2:**
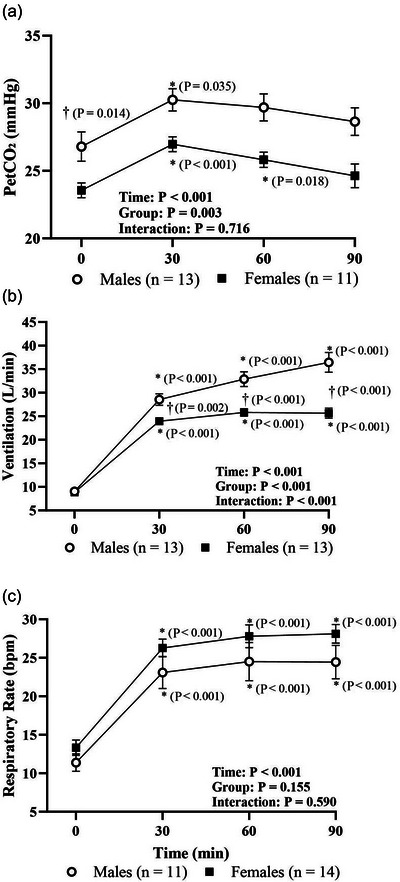
(a) End‐tidal CO_2_ partial pressure ($P_{\mathrm{ETC}O_{2}}$); (b) ventilation; and (c) respiratory rate during the exercise protocol. ^*^Difference from baseline; ^†^difference between groups. Data are presented as means and standard error.

Data on respiratory parameters were acquired for all time points in 26 participants (13♂ 13♀). Figure 2 presents the respiratory markers throughout the experimental period. Time (*P *< 0.001) and group (*P *= 0.003) differences were observed for $P_{\mathrm{ETC}O_{2}}$, with males having a significantly higher $P_{\mathrm{ETC}O_{2}}$ at baseline (*P *= 0.014), with no significant differences at *t*_30_ (*P* = 0.573), *t*_60_ (*P *= 0.559) and *t*_90_ (*P *= 0.741) in comparison with females (Figure 2a). The within‐group analyses revealed that males presented a significant increase at *t*_30_ (*P *= 0.035) in relation to *t*_0_, with subsequent significant decreases at *t*_60_ (*P *= 0.036) in relation to *t*_30_ and *t*_90_ (*P *= 0.047) in relation to *t*_60_. Females significantly increased $P_{\mathrm{ETC}O_{2}}$ at *t*_30_ (*P *< 0.001) and *t*_60_ (*P *= 0.018) in relation to baseline. Significant time (*P *< 0.01), group (*P *< 0.01) and their interaction (*P *< 0.01) were identified regarding ventilation, whereby males presented a higher ventilation than females at *t*_30_ (*P *= 0.002), *t*_60_ (*P *< 0.001) and *t*_90_ (*P *< 0.001). The within‐group analyses revealed that there was a significant increase in ventilation at *t*_30_, *t*_60_ and *t*_90_ (*P *< 0.001) for all time points in relation to baseline in both males and females (Figure 2b). Lastly, significant increases in respiratory rate were found at *t*_30_, *t*_60_ and *t*_90_ (*P *< 0.001 for all time points) in comparison to baseline values in males and females (Figure 2c).

### 3.3 Cerebrovascular and cardiac responses to exercise in the heat

**FIGURE 3:**
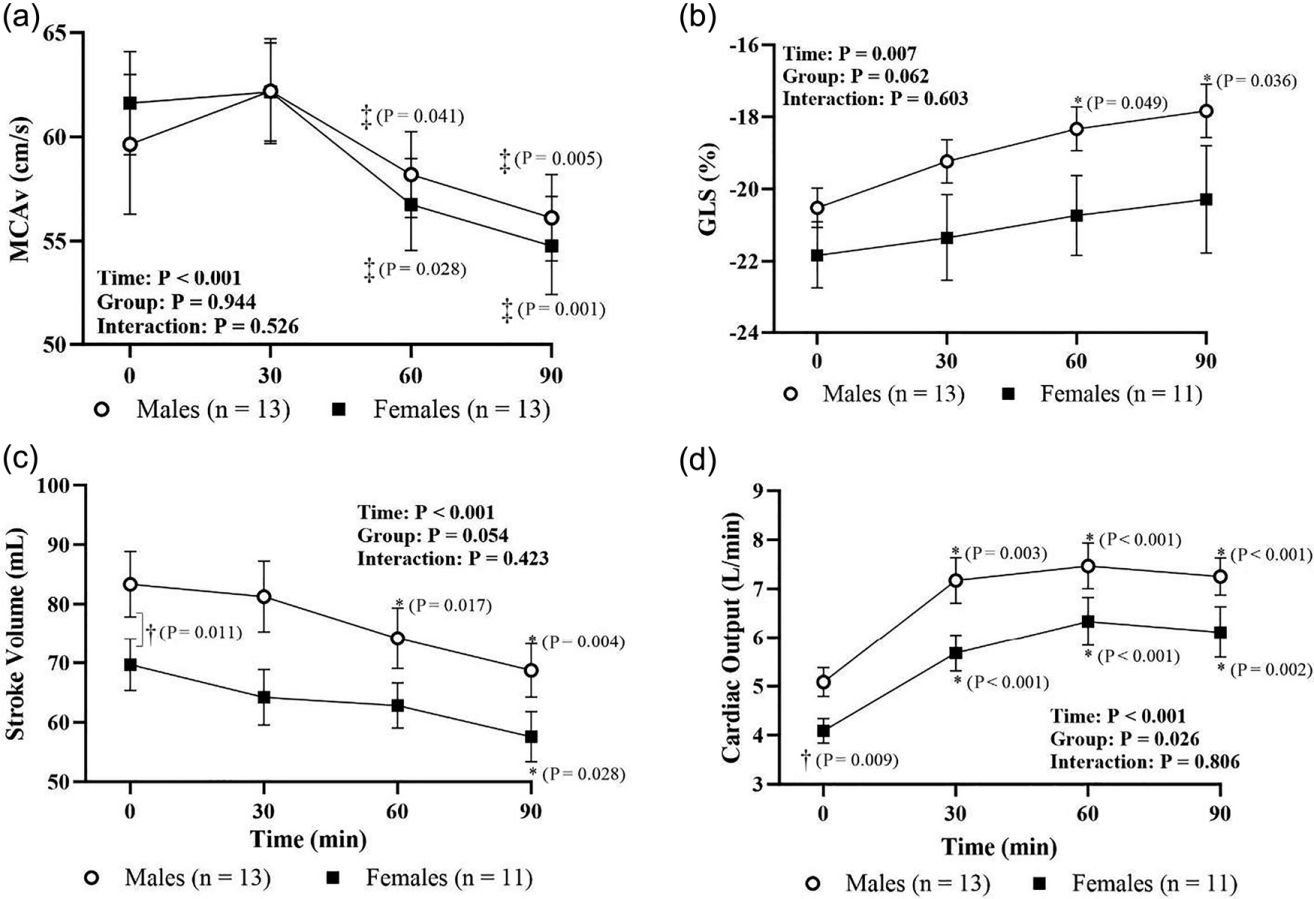
Middle cerebral artery velocity (MCAv) (a), global longitudinal strain (GLS) (b), stroke volume (SV) (c), and cardiac output (*Q̇*) (d) during the exercise protocol. ^*^Difference from baseline; ^†^difference between groups; ^‡^difference in relation to *t*_30_. Data are presented as means and standard error.

Complete cerebrovascular data were collected in 26 participants (13♂ 13♀), with inadequate or poor temporal acoustic windows the main reason for exclusion of the other participants. There was no significant time × group interaction for MCAv (*P* = 0.526) (Figure 3). Indeed, males and females both had similar responses in MCAv, with a non‐significant increase from baseline to *t*_30_ (♂ +4.1%; ♀ +0.87%; *P* = 1.000 and *P* = 1.000, respectively), and significant reductions at *t*_60_ (♂ −6.4%; ♀ −8.7%; *P *= 0.041 and *P *= 0.028, respectively) and *t*_90_ (♂ −9.7%; ♀ −11.88%; *P *= 0.005 and *P *= 0.001, respectively) in relation to *t*_30_, but not baseline (Figure 3a). A similar result was found using changes from baseline instead of absolute values, with a significant time effect (*P *< 0.001), but no significant group effect (*P *= 0.428) or group and time interaction (*P *= 0.694). The within‐group analysis revealed a significant reduction in both males and females at *t*_60_ (*P *= 0.020 and *P *= 0.014, respectively) and *t*_90_ (*P* = 0.002 and *P* < 0.001, respectively) in comparison with *t*_30_, showing a similar result as when analysed using absolute values.

Complete echocardiographic data sets were acquired in 24 participants (13♂ 11♀), with sonographer unavailability or characteristics of the volunteers being the reasons for missing data. Males showed a significantly different GLS (i.e. a less negative value) at *t*_60_ (*P *= 0.049) and *t*_90_ (*P *= 0.025) than at baseline, whereas females did not exhibit any significant differences in this variable relative to baseline levels (Figure 3b). A decrease in SV was observed in males at *t*_60_ and *t*_90_ (*P *= 0.017 and *P *= 0.002, respectively) and in females at *t*_90_ (*P *= 0.028) in relation to baseline (Figure 3c). Males showed a higher SV at *t*_0_ (*P *= 0.011), with no significant sex differences *t*_30_ (*P *= 0.090), *t*_60_ (*P* = 0.971) and *t*_90_ (*P* = 0.823) than females. A group effect was observed for *Q̇* (*P* = 0.026), wherein males presented a significantly higher *Q̇* at baseline (*P *= 0.009). No sex differences were observed at *t*_30_ (*P* = 0.103), *t*_60_ (*P* = 0.859) and *t*_90_ (*P* = 0.745). In addition, *Q̇* increased significantly at *t*_30_ (*P *= 0.003 and *P *< 0.001, respectively), *t*_60_ (*P *< 0.001 and *P *< 0.001, respectively) and *t*_90_ (*P* < 0.001 and *P *= 0.002, respectively) in males and females, respectively, when compared with baseline (Figure 3d).

### 3.4 Relationships between changes in variables

**FIGURE 4:**
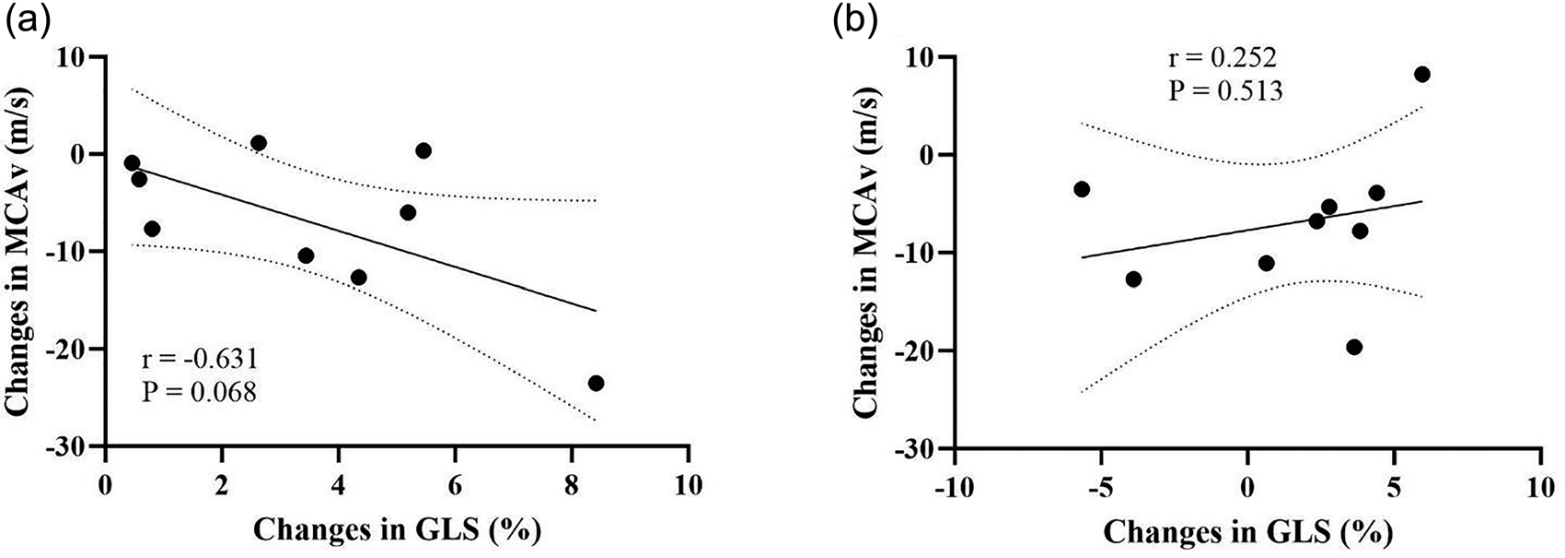
Correlation of changes from baseline to 90 min between middle cerebral artery velocity and global longitudinal strain in (a) males and (b) females. GLS, global longitudinal strain; MCAv, middle cerebral artery velocity.

Correlations relating changes in MCAv and LV GLS from baseline to 90 min in both males and females revealed that, in males, changes in MCAv were correlated with changes in GLS (*r *= −0.631, *P *= 0.068) (Figure 4a). This was not the case in females (*r *= 0.252; *P *= 0.513) (Figure 4b). Changes in *T*_c_ showed strong and significant correlations with HR in both males (*r *= 0.596; *P *= 0.007) and females (*r *= 0.552; *P *= 0.022). Supporting Information.

## 4 DISCUSSION

In the present study, we compared the acute effects of exercise in the heat on MCAv and LV GLS, in healthy male and female volunteers. We observed that, while 90 min of exercise in the heat had similar effects on MCAv, a larger decrease in LV GLS was observed in males. There was also a stronger correlation between changes in GLS and MCAv in males compared to females. These data suggest that, under challenging physiological conditions, LV GLS plays a different role in the mediation of MCAv in males versus females.

The major factors that impact brain perfusion include cerebral metabolism, ventilation (especially $P_{\mathrm{ETC}O_{2}}$), *Q̇* and blood pressure (Ogoh & Ainslie, 2009). The contribution of mechanisms regulating CBF likely differs when exercise in the heat is prolonged, particularly given that *Q̇* is impacted by competing demands between the need for blood to subserve muscle metabolism, versus perfusion of the skin to facilitate thermoregulation. In our study, we demonstrated that after an initial increase, MCAv progressively decreased below baseline levels. Whilst HR progressively increased alongside *T*_c_ during exercise in the heat, SV declined while *Q̇* plateaued. A novel observation was that GLS, an index of subtle changes in systolic function (Stokke et al., 2017), decreased during exercise in the heat.

Previous studies have reported reductions in CBF velocity during prolonged exercise (Nybo & Nielsen, 2001; Périard & Racinais, 2015), and that such reductions are exacerbated by heat stress. Périard & Racinais (2015) assessed MCAv in 11 (10♂ 1♀) cyclists during self‐paced exercise in hot (35°C) and cool (20°C) conditions and demonstrated that, after an initial 10% increase in MCAv in both conditions, MCAv exhibited a decrease that was larger under hot conditions. Likewise Nybo & Nielsen (2001), who assessed MCAv in eight endurance‐trained athletes (all male) during exercise in hot (40°C) and cool (18°C) conditions, showed an exaggerated decrease in MCAv and *Q̇* during exercise in the heat. Sato et al. (2016), who studied nine young males during semi‐supine cycling at 60% of ${\dot{V}}_{O_{2}\mathrm{peak}}$ under 35°C and 25°C (both 40%RH) for 40 min, also reported that during hyperthermic exercise CBF decreased, alongside decreases in *Q̇* and SV. The authors also reported a decrease in $P_{\mathrm{ETC}O_{2}}$ and an increase in ventilation (Sato et al., 2016). These papers, based on relatively small sample sizes and using mostly male participants, concluded that the changes observed in MCAv under hot conditions may be related to changes in ventilation and associated $P_{\mathrm{ETC}O_{2}}$, and/or changes in *Q̇* (Nybo & Nielsen, 2001).

Relatively few previous studies have addressed the potential impact of sex differences on the integrated physiological responses that contribute to cerebral perfusion during exercise in the heat. Our study observed a decrease in MCAv in both males and females, with females presenting a larger (non‐significant) decrease in MCAv than males (♂ 9.8% vs. ♀ 11.8%). We also observed significantly lower values of $P_{\mathrm{ETC}O_{2}}$ and ventilation in females. Rivas et al. (2020), in a study with 22 active individuals (11♂ 11♀) who performed cycling‐based exercise in hot (42°C, 10–60% ramp RH) and cool (24°C, 10% RH) conditions, reported that MCAv was reduced in both males and females during exercise in the heat and also relative to the cool condition, and that females exhibited a larger decrease in MCAv than males (♀ 25% vs ♂ 15%) (Rivas et al., 2020). However, the females had a significant ∼12% higher MCAv than the males at baseline, which the authors proposed was a potential contributor to the differences in the CBF velocity response during subsequent exercise in the heat. The authors also reported that females showed lower values in $P_{\mathrm{ETC}O_{2}}$ and ventilation. They suggested that the larger reduction in MCAv during exercise in the heat they observed in females may be attributed to sex differences in respiratory parameters (mainly $P_{\mathrm{ETC}O_{2}}$), alongside differences in cardiovascular parameters such as blood pressure and HR. Our study, which directly measured these variables, accords with the findings of Rivas et al. (2020), as we found a greater reduction in MCAv in females (even if it was not significantly different), as well as lower values of $P_{\mathrm{ETC}O_{2}}$ and ventilation in females. In contrast, however, there was no significant difference between males and females in MCAv at baseline in our study (♂ 59.64 ± 3.34 vs. ♀ 61.62 ± 2.47 cm s^−1^; *P* = 0.638).

Rivas et al. (2020) speculated that the larger decrease in MCAv that they observed in females could be explained, at least partly, by differences in haemodynamic parameters (blood pressure and HR), because the females had significantly lower blood pressure than males during exercise in the heat. To our knowledge, our study is the first to extend this finding via analysis of LV systolic function (GLS, SV and *Q̇*) in male and female participants. Our novel findings include that males exhibited diminished LV GLS, while females maintained LV GLS during exercise in the heat. In addition, changes in MCAv showed a strong correlation with changes in GLS in males, but not in females. These findings indicate that despite similar reductions in MCAv velocity, males and females may regulate their cerebrovascular responses via different mechanisms.

Despite SV (Rutkowski et al., 2020) and *Q̇* (Argiento et al., 2012) often being reported to be higher in males than females, females appear to have higher myocardial strain (Lawton et al., 2011; Rutkowski et al., 2020; St Pierre et al., 2022). Lawton et al. (2011) studied 60 healthy volunteers (♂28 ♀32) at rest using MRI‐based multiparametric analysis, and reported that females have approximately 14% higher GLS (i.e. greater deformation) than males. In addition, significantly larger global circumferential strain (GCS) was observed in females (Lawton et al., 2011). The study made measurements at rest, in supine subjects who undertook breath‐holding manoeuvres. Rutkowski et al. (2020) studied 49 healthy individuals (♂20 ♀19) using cardiac four‐dimensional flow and two‐dimensional cine‐MRI, obtaining indexes related to velocity‐based metrics of flow, kinetic energy, vorticity, efficiency as well as cardiac strain. They reported that LV vorticity and GLS, GCS and radial strain were higher in females than males. They also identified a moderate correlation between LV strain and vorticity. In keeping with the reports above, these findings indicate that, at rest, females and males appear to have distinct cardiac functional parameters. Our findings extend these studies in that we collected data on LV systolic function during exercise in the heat. Our observation that males had a decrease in myocardial deformation (less negative GLS), while females exhibited preserved GLS, is consistent with previous data collected in resting individuals.

The present study has several limitations. We did not include a room temperature exercise condition as a comparator. However, evidence in the literature has consistently shown that CBF is largely reduced during exercise in the heat in comparison with exercise in cooler conditions (Périard & Racinais, 2015; Rivas et al., 2020; Sato et al., 2016). Also, factors other than cardiac function (e.g. $P_{\mathrm{ETC}O_{2}}$, CO_2_ reactivity, cerebral autoregulation and neurovascular coupling) may have contributed to sex differences in MCAv, as suggested in studies discussed above. But, to our knowledge, this is the first study to investigate sex differences in MCAv and its relation to changes in LV function, providing a novel insight into the influence of cardiac control on cerebrovascular function. Baseline differences between males and females, for example, in fitness and/or body composition, may have impacted MCAv responses to our intervention. Our aim was to perform an ecologically valid experiment where males and females were exposed to the same task requirements. This study design is relevant in military and workplace (e.g. mining, construction) contexts, where baseline differences cannot always be mitigated. Another limitation is the impact of posture on the responses that we assessed. We performed echocardiographic assessments in a semi‐recumbent position to optimise image quality, whereas participants were upright when they walked on the treadmill, which may have impacted our systolic function outcomes to some degree. Nonetheless, our assessments are more ecologically valid than those collected in previous MRI‐based experiments, which were performed in a fully recumbent posture involving breath‐holding manipulations. Our echocardiographic assessments took approximately 2.5 min to complete. Previous evidence suggests that myocardial deformation remains lower related to baseline values for 5 min after exercise performance (Locatelli *et al.*, 2024). The impact of our brief breaks to assess GLS should have therefore minimally impacted our results. The small age difference between males and females (♂ 25.74 ± 3.62 vs. ♀ 30.00 ± 6.26 years; *P *= 0.016) may also have contributed to the differences that we observed. However, it would be expected that older individuals, in this case females, would exhibit impaired systolic function, whereas the opposite was the case in our study. Finally, we did not control for contraceptive medication or phases of the menstrual cycle, as the aim of the study was to conduct an ecologically valid experiment; it is not possible in military, industrial or workplace settings to avoid exercise and environmental exposures based on use of the pill or phase of the cycle.

### 4.1 Conclusion

This is the first study that has reported systolic function (GLS, SV, *Q̇*) and its relationship to cerebrovascular function in males and females in response to a physiological challenge in humans under a heated environment. In addition, we employed LV GLS to reflect cardiac function, which has not been undertaken in this type of exercise setting before. Although males and females exhibited a similar response in terms of MCAv when they exercised in the heat, the males had a decline in LV GLS, whereas female LV GLS was relatively preserved. This evidence suggests that sex differences may exist in the regulation of cerebrovascular function during exercise in the heat in humans. Future studies are warranted to investigate sex differences in cerebrovascular responses to exercise in environmentally challenging conditions, including those undertaken with a broader age range and in individuals with various cardiovascular diseases, and expanding the investigation to cerebrovascular and systolic function responses during the recovery period after exercise in the heat.

## AUTHOR CONTRIBUTIONS

João Carlos Locatelli: formal analysis, investigation, visualization, writing—original draft preparation; Juliene G. Costa: investigation, visualization, writing—review and editing; Kristanti W. Wigati: investigation, visualization, writing—review and editing; Jesse Criddle: investigation, visualization, writing—review and editing; Julie Collis: investigation, visualization, writing—review and editing; Xingwei Xu: investigation, visualization, writing—review and editing; Louise Naylor: conceptualisation, investigation, supervision, writing—review and editing; Howard H. Carter: conceptualisation, investigation, methodology, project administration, supervision, investigation, writing—review and editing; Shane K. Maloney: visualisation, writing—review and editing; Robert A. McLaughlin: visualization, writing—review and editing; Andrew Haynes: supervision, investigation, writing—review and editing; Helen Jones: funding acquisition, visualization, writing—review and editing, Keith George: funding acquisition, conceptualization, writing—review and editing; Daniel Green: Conceptualization, funding acquisition, methodology, resources, visualisation, supervision, writing—original draft preparation, writing—review and editing. All authors have read and approved the final version of this manuscript and agree to be accountable for all aspects of the work in ensuring that questions related to the accuracy or integrity of any part of the work are appropriately investigated and resolved. All persons designated as authors qualify for authorship, and all those who qualify for authorship are listed.

## CONFLICT OF INTEREST

R.A. McLaughlin is a co‐founder and Director of Miniprobes Pty Ltd, a company that developes optical imaging systems. Miniprobes Pty Ltd did not contribute to or participate in this study. The remaining authors declare they have no conflicts of interest.

## Supporting information



Supporting Information.

## Data Availability

The authors declare that the raw data that support the findings of this study are available upon request.
